# Targeting therapy effects of composite hyaluronic acid/chitosan nanosystems containing inclusion complexes

**DOI:** 10.1080/10717544.2022.2112995

**Published:** 2022-08-18

**Authors:** Zhiwei Liang, Dongmei Chen

**Affiliations:** a National Reference Laboratory of Veterinary Drug Residues (HZAU) and MAO Key Laboratory for Detection of Veterinary Drug Residues, Wuhan, Hubei, China; b MOA Laboratory for Risk Assessment of Quality and Safety of Livestock and Poultry Products, Huazhong Agricultural University, Wuhan, Hubei, China

**Keywords:** *Staphylococcus aureus*, enrofloxacin, composite nanosystems, pharmacokinetics, therapy effects

## Abstract

In order to solve the difficulties in the treatment of *Staphylococcus aureus* infections, a novel enrofloxacin-cyclodextrin (β-CD) inclusion complexes (IC) containing hyaluronic acid/chitosan (HA/CS) self-assemble composite nanosystems covered by poloxamer 188 was designed in our previous study. In this study, the sustained release peforemance, targeting delivery, and therapy effects of the enrofloxacin-composite nanosystems were evaluated *in vivo*. The enrofloxacin-composite nanosystems had uniform size and smooth surface with drug loading capacity (LC) of 9.92 ± 0.3%. Thermogravimetric analysis (TGA) showed that the material used for the preparation of the enrofloxacin-composite nanosystems did not affect the thermal stability of enrofloxacin. Compared with enrofloxacin injection and enrofloxacin polymeric nanoparticles, the enrofloxacin-composite nanosystems had excellent sustained-release performance *in vivo*. The enrofloxacin-composite nanosystems have specific targeting to the infection site of *Staphylococcus aureus*. The excellent sustained release and targeting delivery properties ensure that the anti-infective treatment effect of the enrofloxacin-composite nanosystems *in vivo* was higher than that of enrofloxacin injection and enrofloxacin polymeric nanoparticles. It can more effectively promote the wound healing. These results suggest that our previous designed enrofloxacin-composite nanosystems will be a promising formulation for effective targeting therapy of *Staphylococcus aureus* infections.

## 1. Introduction

*Staphylococcus aureus* (*S. aureus*) is an important zoonotic pathogen, which can not only cause huge economic losses to the animal husbandry industry all over the world by parasitizing animals, but also spread to humans through direct contact with animals or contaminated food, seriously threatening human health (Kim et al., 2018; Moreno-Grúa et al., 2020). *S. aureus* mainly colonizes the skin and mucous membranes of the host and clinically causes a variety of diseases, such as skin infections, abscesses, impetigo, necrotizing pneumonia, sepsis, atherosclerosis, and osteomyelitis (Zhou et al., 2012). In the livestock industry, approximately 380 tons of milk are lost globally each year due to *S. aureus* infections (Loiselle et al., 2009). Some virulence factors secreted by *S. aureus* cause foodborne illness in humans. The U.S. Centers for Disease Control estimates that 240,000 cases of staphylococcal food poisoning occur each year, resulting in 1,000 hospitalizations and 6 deaths (Schelin et al., 2017).

At present, antibiotics are mainly used to treat *S.aureus* infection in clinic. Enrofloxacin, the first fluoroquinolone approved for veterinary use, is used to treat animal infections caused by a variety of gram-positive bacteria due to its broad-spectrum antibacterial activity and broad distribution in the body, which include methicillin-resistant *S. aureus* (MRSA) and methicillin-susceptible *S. aureus* (MSSA) (Hauschild et al., 2012; Pei et al., 2020). Although the therapeutic efficacy of enrofloxacin has been fully recognized, the rapid metabolism limit its clinical appilcation. In order to maintain its blood concentration, it is often necessary to increase the dosage frequency. However, long-term high-dose and high-frequency use will lead to some obvious side effects and severe drug resistance. In addition, the nonspecific distribution of enrofloxacin *in vivo* also limits its clinical application. Therefore, there is an urgent need to develop an intelligent delivery system that can improve the solubility, sustained release, and targeting of enrofloxacin to achieve precise treatment of *S. aureus* infection sites *in vivo*.

In recent years, with the continuous advancement of nanotechnology, nanogels have been shown to be inert due to their good physicochemical properties (controlled drug release and affinity for aqueous solutions), excellent colloidal stability, high cellular internalization and the tendency to remain inert in the blood (Yan et al., 2019; Akbarzadeh et al., 2021). Furthermore, due to the special properties of materials, nanogels can respond to bacterial infection microenvironments (e.g. lower pH, overexpressed enzymes and toxins, etc.) (Chiriac et al., 2019). For example, red blood cell (RBC) nanogels have been shown to neutralize MRSA-related toxins in the extracellular milieu and stimulate macrophages to engulf bacteria (Zhang et al., 2017). Among the numerous materials, negatively charged hyaluronic acid (HA) has been widely used in the design of nanogels due to its good biocompatibility and responsiveness to hyaluronidase secreted by bacteria. For example, the on-demand release of antibacterial agents from polymer-hyaluronic acid nanoparticles can effectively kill MRSA (Yuan et al., 2021). However, compared with single responsiveness, dual responsiveness is more conducive to the delivery of target sites (Banstola et al., 2021). As a natural biodegradable polymer, chitosan (CS) has a proton sponge effect corresponding to acidic conditions, and at the same time, its positive charge properties make it also have good antibacterial activity, which can synergistically enhance the effect of antibacterial agents (Kurdtabar et al., 2018; Gadkari et al., 2019). For example, the chitosan cinnamaldehyde nanoparticles prepared by Rahul et al. have synergistic antibacterial activity against Gram-positive and Gram-negative bacteria (Gadkari et al., 2019). However, both nanogels suffer from relatively fast and premature release.

Based on this, our group previously designed a pH/hyaluronidase dual responsive enrofloxacin-cyclodextrin (β-CD) inclusion complexes (IC) containing HA/CS self-assemble composite nanosystems covered by poloxamer 188 for targeted ‘on-demand’ delivery. The novel targeted ‘on-demand’ delivery composite nanosystems combine the triple controlled release of cyclodextrin (β-CD) inclusion complexes, poloxamer 188 encapsulation and nanogels, as well as the responsiveness release of HA and CS (Liu et al., 2021). Our previous study demonstrated that the clever dispersion of enrofloxacin inclusion complexes into HA/CS nanogels covered by poloxamer 188 can achieve bacterial-targeted responsive release in the acid medium, hyaluronidase containing medium, and LB broth medium where *S. aureus* present (Liu et al., 2021). It had stronger sustained release than the polymeric nanoparticles formulated by entrapping of IC into poloxamer 188 and the single HA/CS nanogels. In this study, the pharmacokinetics, targeting distribution and anti-infective effects of the composite nanosystems were systematically studied *in vivo*.

## 2. Materials and methods

### 2.1. Materials

HA (MW: 403.31, content: 97%) was purchased from Macklin (Shanghai, China). Hydroxypropyl methyl cellulose (HPMC, Viscosity: 50–20,000 mPa.s) was obtained from Tianjin guangfu fine chemical research institute. CS (MW:161.16 kDa, viscosity: 50–800 mPa.s, degree of acetylation 80–95%), sodium tripolyphosphate (TPP, MW: 367.86), β-cyclodextrin (β-CD, MW: 1134.98), sodium hydroxide, formic acid and acetic acid were purchased from Sinopharm group chemical reagent co. Ltd. Ethyl alcohol (95%) was bought from Wuhan xingheda trading co. Ltd. Poloxamer 188 were purchased from sigma-aldrich, USA. Methanol (HPLC) was purchased from Grace chemical technology co., LTD. Acetonitrile was bought from Sigma-Alorich. Sodium chloride (NaCl, ≥99.5%) were purchased from Wuxi jiani chemical co. Ltd. Ultrapure water was prepared by a Microporous Purification® Purified Water System (Milli-Q Ltd., France). All other reagents not special emphasis were of analytical pure.

### 2.2. Sources of strains and animals

S.*aureus* (ShangHai-01): a clinical isolate from the National Veterinary Drug Residues Benchmark Laboratory of Huazhong Agricultural University.

Healthy 6–8 week old male Balb/c mice (SPF, 18 ± 2 g) and nude mice were provided by Laboratory Animal Center of Huazhong Agricultural University (HAZU) (Wuhan, China). All the animal experiments were performed in accordance with the approval of the Animal Care and Use Committee of Huazhong Agricultural University (Wuhan, China).

### 2.3. Preparation of enrofloxacin-composite nanosystems

Enrofloxacin-composite nanosystems were prepared according to our previous report (Liu et al., 2021). In short, 2 g of enrofloxacin and 12 g of β-cyclodextrin were dissolved in 30 mL of 0.1 mol/L NaOH, and stirred at 1500 r/min at 80 °C for 2 h. Then, 30 mL solution of 10% poloxamer 188 and 1.6% HA was directly added into the above solution under stirring at 1000 r/min. The stirring was continued until the liquid became milky white. Subsequently, 20 mL of 0.5% acetic acid solution containing 2.4% CS was added dropwise into the above milky white solution. The mixture was stirred for 15 min after addition of CS solution, and 1 mL of an aqueous solution containing 16% TPP was added dropwise to the above solution, and stirred at a constant speed for 2 h to form a stable composite nanosystems. Finally, the volume of the composite nanosystem was kept at 80 mL.

In order to use as control, the simple polymeric nanoparticles containing IC was also prepared as the above-mentioned process without adding of HA and CS solutions.

### 2.4. Characterization of enrofloxacin-composite nanosystems

#### 2.4.1. Particle size, Zeta potential and polydispersity index (PDI)

The size, PDI and Zeta potential of the composite nanosystems were determined by using a Zetasizer ZX3600 (Malvern Instruments, UK) at 25 °C. Prior to the determination, the nanosystems were diluted to 2.5 mg/ml to obtain the best kilo count of 20–400 per second for measurement. All the measurements were repeated 3 times by using samples from independent batches.

#### 2.4.2. Transmission electron microscopy (TEM)

The composite nanosystems was diluted to 2.5 mg/mL, and after negative staining, 2 μL of the sample was drawn and dropped onto a copper grid with a thin film, and its microscopic morphology was examined by transmission electron microscopy (HITACHI, Japan) after drying in the room.

#### 2.4.3. Encapsulation efficiency and loading capacity

First, the prepared enrofloxacin-composite nanosystems was centrifuged at 14,000 r/min (Hitachi Centrifugation CR21GIII; Hitachi Koki Co., Ltd., Japan) at 4 °C for 60 min, and the precipitate and supernatant were collected respectively. The enrofloxacin content in the supernatant after centrifugation was determined by high performance liquid chromatography (HPLC) to determine encapsulation efficiency (EE). The nanosystem pellets were lyophilized for 48 h (freeze drying system; Labconco, USA) to test loading capacity (LC). The 10 mg of freeze-dried nanosystems were dissolved in 10 mL of mobile phase, and the nanosystems were disrupted by using a cell sonicator, followed by centrifugation for 10 min. The drug in the supernatant was analyzed by HPLC after filtration. EE and LC are defined as follows:

EE (%) = [(enrofloxacin addition amount − enrofloxacin weight in supernatant)/(enrofloxacin addition amount)] × 100%

LC (%) = [(weight of enrofloxacin in preparation)/(weight of preparation)] × 100%

#### 2.4.4. Thermogravimetric analysis

Thermogravimetric analysis (TGA) determines the mass of a substance as a function of temperature or time under programmed temperature control. The thermal stability of all samples was measured by a thermogravimetric analyzer (TG 209 C, NETZSCH, Germany). The sample mass was 5–10 mg, the measurement temperature was ranged from room temperature to 500 °C, and the heating rate was 10 °C/min.

### 2.5. Cytotoxicity

The cytotoxicity of the composite nanosystems was determined by MTT method. RAW264.7 cells were added into a 96-well plate at a density of 10^4^ cells per well. After culturing for 24 h at 37 °C in a 5% CO_2_ environment, the cells were completely adherent. The medium in the culture plate was aspirated and discard, 200 μL different concentrations (32 μg/mL, 16 μg/mL, 8 μg/mL, 4 μg/mL, 2 μg/mL, calculated as the concentration of encapsulated enrofloxacin) of composite nanosystems was added into each well, the control group was only added the medium without composite nanosystems, and 3 parallels were set for each concentration. After that the culture was continued for 24 h, MTT (5 mg/mL) was added and used for measurement. The control group has been calculated 100%. The sample calculation formula is as follows:

Cell viability (%) = (sample OD value/control OD value) × 100%

### 2.6. Pharmacokinetics

#### 2.6.1. Animal studies

Twelve male SD rats were fasted and had free access to water for 12 h before administration. Rats were randomly divided into three groups and each group contained 4 rats. The rats were intravenously injected with enrofloxacin injection, enrofloxacin polymeric nanoparticles and composite nanosystems at an equivalent enrofloxacin dose of 2.5 mg/kg. After intravenous administration of 0.25, 0.5, 1, 2, 4, 6, 8, 12, 24, 48 and 72 h, 0.2 mL blood was collected from the heart and placed into the heparinized tubes. The samples were centrifuged at 5000 r/min for 10 min to obtain the plasma for content detection by HPLC. The pharmacokinetic parameters were fitted by WinNonLin software.

#### 2.6.2. HPLC

The 100 μL of the rat plasma was took into a 2 mL centrifuge tube. Then, 200 μL of methanol was added into the centrifuge tube. The solution was mixed and vortex for 1 min, sonicated for 5 min, centrifuged at 14,000 r/min for 10 min, passed through a 0.22 μm filter membrane for test. The fluorescence detector (HPLC-FLD) method was used. Chromatographic column: Waters C18 column (4.6*250 mm, 5 μm), excitation wavelength: 287 nm, emission wavelength: 450 nm. Mobile phase: phase A of 0.1% aqueous formic acid: phase B of acetonitrile = 82:18, retention time: 12 min.

### 2.7. Targeting delivery performance of enrofloxacin-composite nanosystems

Tetrakis (4-carboxyphenyl) porphine was encapsulated into the composite nanosystems as a fluorescent probe. A mouse wound infection model was established to test the targeting delivery performance of the composite nanosystems. Briefly, the nude mice were anesthetized with ether cotton balls and the epidermis was cut on the right hindlimb by surgical scissors to form a 5 mm diameter wound. Subsequently, *S. aureus* was injected into the wound (10^7^ CFU/mL, 100 μL). After infection for 24 h, the same amount of free fluorescent probe and composite nanosystems loaded fluorescent probe were injected intravenously (*n* = 3). After medication, the fluorescence intensity at the infection site was observed at a given time (6, 12, 24, and 48 h) by the IVIS Spectrum small animal *in vivo* imaging system (PerkinElmer, USA). At the end of the experiment, the fluorescence of the viscera was measured after the mice was slaughtered.

### 2.8. Anti-infection efficacy evaluation

BALB/C mice were anesthetized with ether cotton balls, and the epidermis was cut on the right hindlimb by surgical scissors to form a 5 mm diameter. The wound of BALB/C mice was infected with 0.1 mL of 10^7^ CFU/mL ShangHai-01 bacterial solution for 1 day, and then divided into 4 groups of 6 mice, namely enrofloxacin injection, enrofloxacin nanoparticles, composite nanosystem group (measured by enrofloxacin, 5 mg/kg.bw) and nontreatment control group. It was administered intravenously for three consecutive days, once a day. The wound size was observed and recorded every day. In each group, 3 mice were sacrificed at the end of the administration and on the 3rd day after the last administration. The tissue of the wound infection site was taken after execution and homogenized with PBS. After dilution to a certain number of times, bacterial plate counts were then performed.

### 2.9. Statistical analysis

Data are presented as mean ± standard deviation (SD) and analyzed by the SPSS software (version 20, IBM, New York, USA). Statistical significance was defined as a p-value of 0.05 by the one-way ANOVA.

## 3. Results and discussion

### 3.1. Characterization of enrofloxacin-composite nanosystems

**Figure 1.**
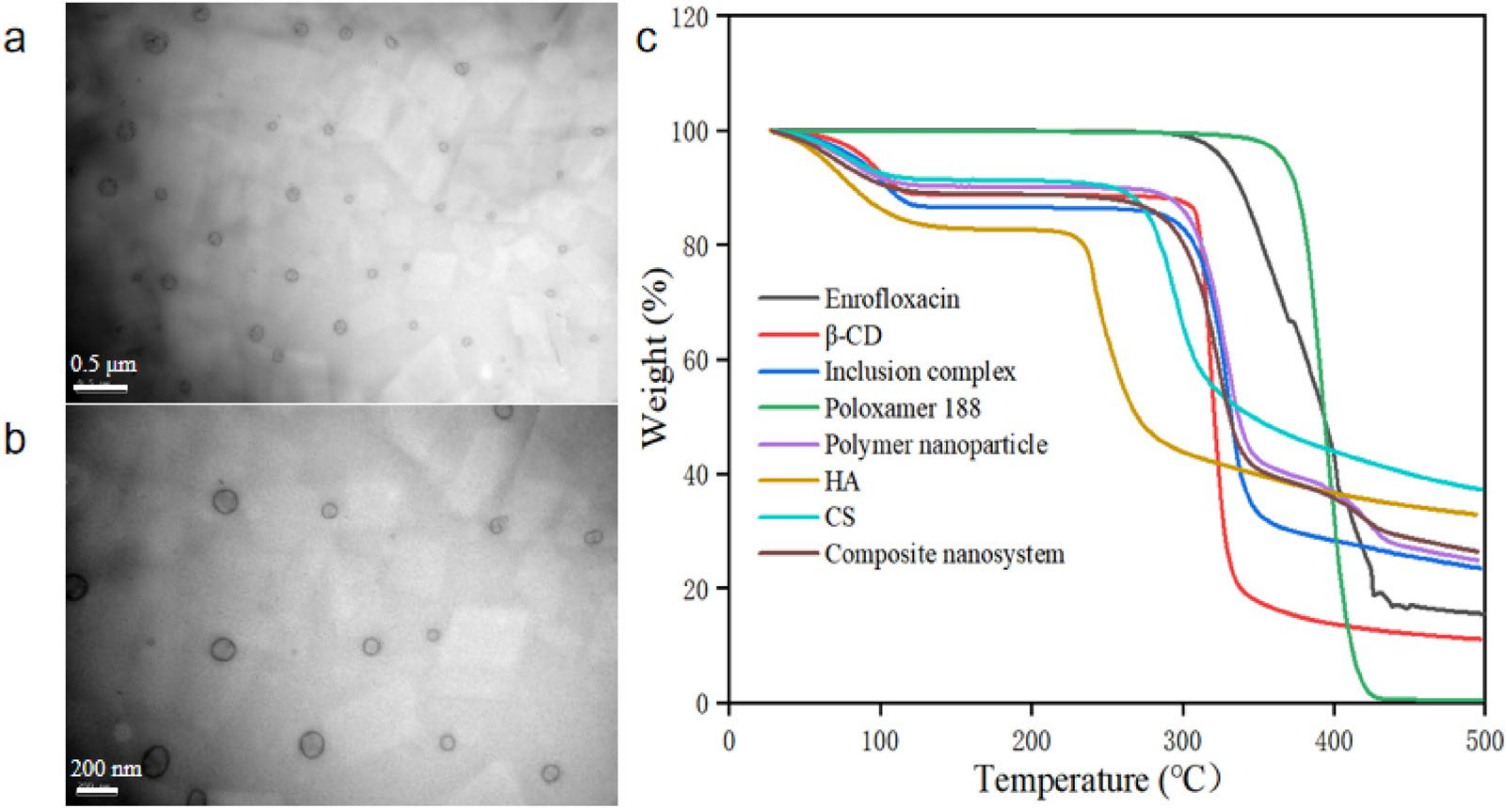
Characterization of enrofloxacin-composite nanosystem. Both (a) Bar = 0.5 μm and (b) Bar = 200 nm are TEM images of enrofloxacin-composite nanosystems. (c) DSC of the enrofloxacin-composite nanosystems and individual components.

The enrofloxacin-composite nanosystems were homogeneous white suspensions with an average hydrodynamic size of 116.3 ± 17.7 nm and PDI of 0.26 ± 0.01 (Table 1). It has been reported that nanoparticles <10 nm are easily metabolized and excreted by the kidney, while nanoparticles >200 nm can be quickly phagocytosed by the mononuclear phagocyte system (MSP) and accumulate in the liver and spleen, resulting in lower drug content at the site of infection (Zhang et al., 2020). Therefore, the size of the enrofloxacin-composite nanosystems might be beneficial for its long circulation *in vivo*. TEM images showed that the nanosystems were transparent spherical with smooth surface and uniform particle size, and the particle size determined by TEM was smaller than that measured by DLS (Figure 1a and 1b). This may be because that the size detected by DLS was in the aqueous state and the size was the diameter of hydration, all free water and even some hydrated water is evaporated while in the case of TEM sample preparation. This phenomenon is consistent with our previous reports (Algharib et al., 2020; Liu et al., 2021). LC and EE are indispensable parameters for drug delivery system. The EE and LC of enrofloxacin-composite nanosystems were 93.5 ± 2.9% and 9.92 ± 0.3%, respectively. The high LC will help to reduce the amount of clinical medication required, thus avoiding unwanted side effects. The zeta potential was −3.30 ± 0.4 mV. Although, zeta potential values other than −30 mV to +30 mV are generally considered to have sufficient repulsive forces for better physical colloid stability, conversely, smaller zeta potentials lead to particle aggregation and flocculation due to van der Waals attraction (Jeong et al., 2021). However, non-zero Zeta potential values can stabilize the system by imparting sufficient electrostatic stability between particles (Jeong et al., 2018). In addition, a certain thickness of the Poloxamer 188 layer not only has excellent function of inhibiting the crystallization of guest molecules, but also can effectively stabilize the system (Xie et al., 2008; Liu et al., 2021). Thermogravimetric analysis of enrofloxacin-composite nanosystems and their components was performed at 10 °C/min under nitrogen atmosphere from room temperature to 500 °C. As shown in Figure 1(c), enrofloxacin-composite nanosystems, polymeric nanoparticles, IC, β-cyclodextrin, CS and HA were all about 10% mass loss between room temperature and 100 °C, which may be due to the loss of residual moisture in these substances (Chuysinuan et al., 2020; Huang et al., 2020; Eddarai et al., 2022). Subsequently, the enrofloxacin-composite nanosystems suffered a 60% mass loss at 300–350 °C, which may be due to the degradation of the polysaccharide residues of CS and the decomposition of β-CD and HA. When the temperature was increased to 450 °C, the mass of the enrofloxacin-composite nanosystems was lost another 10%, which may be attributed to the degradation of poloxamer 188 and enrofloxacin. This indicates that each material did not significantly affect the thermal stability of enrofloxacin.

**Table 1.** Properties of enrofloxacin-composite nanosystem.

Size(nm)	Zeta potential(mV)	PDI	EE(%)	LC(%)
116.3 ± 17.7	−3.30 ± 0.4	0.26 ± 0.01	93.5 ± 2.9	9.92 ± 0.3

### 3.2. Cytotoxicity

**Figure 2.**
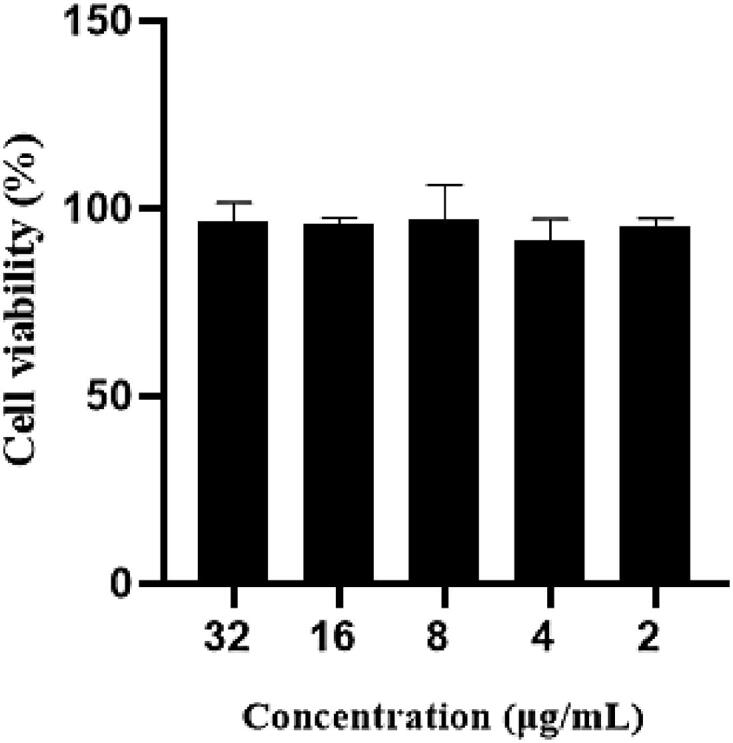
Cytotoxicity of enrofloxacin-composite nanosystem.

The cytotoxicity of enrofloxacin composite nanosystems was documented by MTT method. The cell viability of RAW 264.7 cells was higher than 90% after incubution of different concentrations (2–32 μg/mL) of the composite nanosystem for 24 h (Figure 2), which revealed that the enrofloxacin composite nanosystem has no obvious toxicity to RAW 264.7 cells and will have good safety.

### 3.3. Pharmacokinetics

**Figure 3.**
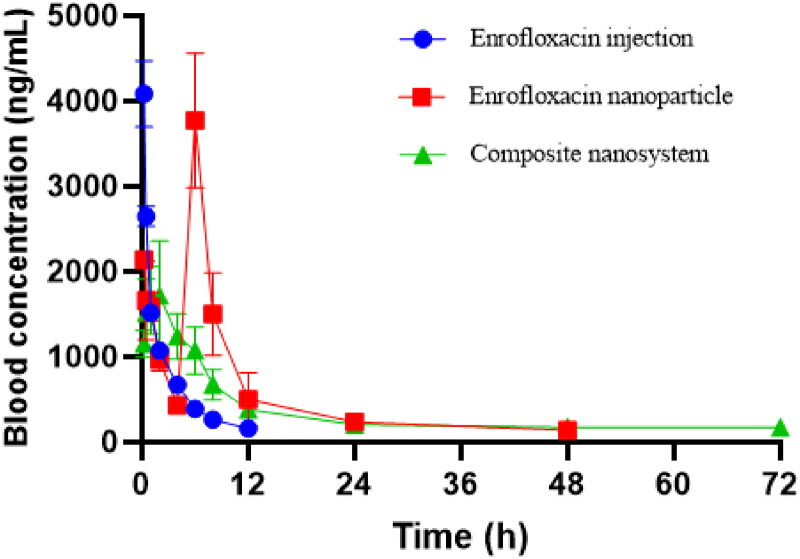
Drug time curve of enrofloxacin-composite nanosystem, enrofloxacin-polymeric nanoparticle and enrofloxacin injection solution.

The plasma enrofloxacin concentration-time curves are shown in Figure 3. After intravenous injection, the plasma concentration of enrofloxacin injection decreased rapidly and was below the detection limit at 12 h. The plasma enrofloxacin concentration-time curves of enrofloxacin-polymeric nanoparticles was gradually decreased with time, and decreased to 0.43 μg/mL at 4 h, but rapidly increased to 3.77 μg/mL at 6 h, subsequently dropped rapidly and below the detection limit within 72 h. This first high concentration might be due to the unencapsulated drug and the burst release, while the second high drug concentration might be due to the degradation or destruction of the polymeric nanoparticles. Although enrofloxacin polymeric nanoparticles have a certain sustained release effect *in vivo*, the second high concentration at 6 h suggests that the single polymeric nanoparticles are unstable *in vivo*, and there may be a risk of early and burst release. Compared to the enrofloxacin polymeric nanoparticles, the enrofloxacin-composite nanosystems exhibited excellent and stable sustained-release properties *in vivo*. The plasma concentration of enrofloxacin nanosystems slowly increased with time, reaching a peak value of 1.72 μg/mL at 4 h, and then decreased slowly and below the detection limit at 96 h. This indicates that the composite nanosystems did not have transient degradation phenomenon, which can effectively avoid the premature release. The triple effect of inclusion complex, nanogels and poloxamer 188 coating can achieve the ideal sustained release. The pharmacokinetic parameters after intravenous injection of enrofloxacin injection, polymeric nanoparticles and enrofloxacin-composite nanosystems are shown in Table 2. The AUC_0-t_, C_max_ and MRT of enrofloxacin injection were 7.86 ± 0.30 h*μg/mL, 4.09 ± 0.32 μg/mL and 3.29 ± 0.05 h, respectively. The AUC_0-t_, Cmax and MRT of enrofloxacin polymeric nanoparticles were 25.41 ± 3.12 h*μg/mL, 3.77 ± 0.64 μg/mL and 12.75 ± 0.39 h, respectively. The AUC_0-t_, Cmax and MRT of enrofloxacin-composite nanosystems were 32.11 ± 3.91 h*μg/mL, 2.08 ± 0.12 μg/mL and 21.49 ± 0.72 h, respectively. The above pharmacokinetic parameters also indicated that enrofloxacin-composite nanosystems had a significant sustained release performance compared with enrofloxacin injection and enrofloxacin polymeric nanoparticles.

**Table 2.** Pharmacokinetic parameters of different enrofloxacin formulations (Mean ± SD, *n* = 4).

Pharmacokinetic parameters	Injection	Polymeric nanoparticles	Composite nanosystems
Ke (1/h)	0.18 ± 0.06	0.04 ± 0.01	0.03 ± 0.01
T1/2 (h)	4.15 ± 1.43	17.77 ± 5.72	31.43 ± 10.89
Cmax (μg/mL)	4.09 ± 0.32	3.77 ± 0.64	2.08 ± 0.12
AUC_0-t_ (h*μg/mL)	7.86 ± 0.30	25.41 ± 3.12	32.11 ± 3.91
MRTlast (h)	3.29 ± 0.05	12.75 ± 0.39	21.49 ± 0.72

### 3.4. Targeting delivery performance of enrofloxacin-composite nanosystem

**Figure 4.**
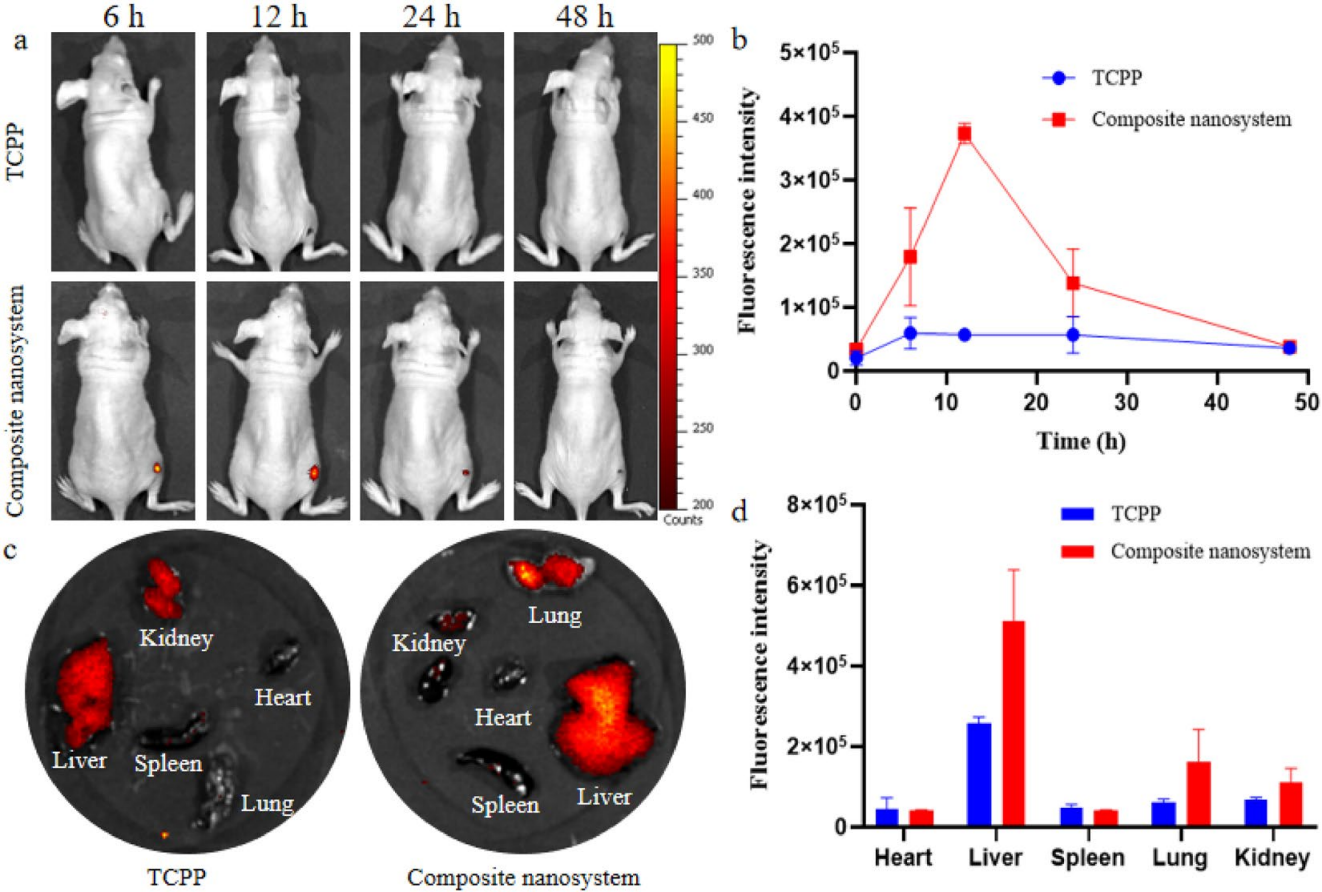
*In vivo* targeting performance of composite nanosystems. (a, b) Fluorescence maps and fluorescence quantitative analysis of *S. aureus*-infected mice following tail vein injection with 100 μL of TCPP and composite nanosystem. (c, d) Fluorescence images and fluorescence quantitative analysis of mouse organs after 2 days of tail vein administration (TCPP and composite nanosystems). In the figure, (a) and (c) are selected from three independent samples. Error bars represent standard deviation determined from three independent measurements.

In view of the excellent targeting delivery of enrofloxacin-composite nanosystems to *S. aureus in vitro* (Liu et al., 2021), the *in vivo* targeting performance to *S. aureus* infection sites was evaluated by establishing a mouse wound infection model. Equal doses of fluorescentprobe-composite nanosystems and fluorescent probe molecules were administered by tail vein injection. It was found that the free fluorescent probe did not specifically target the site of *S. aureus* infection (Figure 4a and 4b). Compared with free fluorescent molecules, the composite nanosystems encapsulated fluorescent molecules can specifically target the infection site of *S.aureus*. During 0 and 12 h, the fluorescence intensity of the composite nanosystems loaded fluorescence probe at the infection site was gradually increased. The fluorescence intensity reached the highest value at 12 h, which was 6.55 times higher than the intensity of free fluorescent molecules. Its fluorescence disappeared at the infected site after 2 d of administration. The targeting of the composite nanosystems to the infection site may be attributed to the responsiveness of HA and CS to the microenvironment of infection site (Ji et al., 2016; Gadkari et al., 2019; Yuan et al., 2021). Two days after the last administration, the fluorescence of composite nanosystems mainly appeared in the liver, lung and kidney (Figure 4c and 4d). At the same time, the distribution of free fluorescent molecules in organs was also much lower than that of the composite nanosystems, which may be due to its rapid clearance *in vivo*. In addition, liver, lung and kidney are the main organs for metabolism and excretion in the body, which also indicates that the nanosystem can be cleared from mice with good safety.

### 3.5. Therapy effects of composite nanosystems

**Figure 5.**
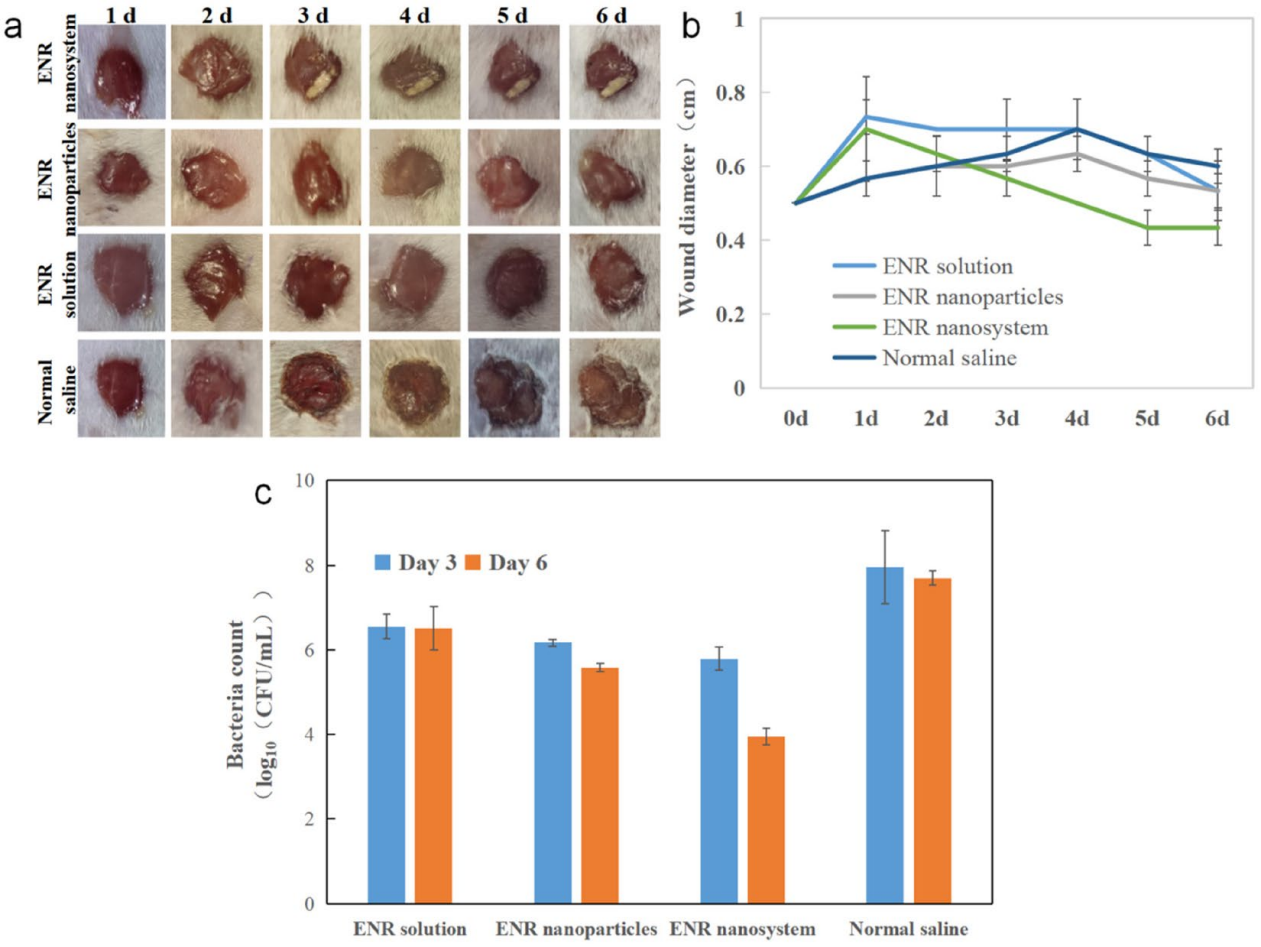
Therapy effects of different enrofloxacin formulations. (a) Representative recovery photographs of enrofloxacin solution, enrofloxacin polymeric nanoparticles, and enrofloxacin-composite nanosystems, and saline treatment over time. (b) Wound recovery diameter over time in enrofloxacin solution, enrofloxacin polymer nanoparticles and enrofloxacin composite nanosystem and saline treatment over time. (c) Bacterial counts at the wound site after 3 and 6 days treatment with enrofloxacin solution, enrofloxacin polymeric nanoparticles, enrofloxacin-composite nanogel and saline. In the figure, (a) and (c) are selected from three independent samples. Error bars represent standard deviation determined from three independent measurements.

Since *S.aureus* frequently colonizes the skin and mucous membranes of the host and is the most common pathogenic microorganism responsible for the development of wound-related infections, the therapeutic effect *in vivo* was evaluated through the mouse wound infection model. The representative diagram of the wound healing process of the infected site of mice is shown in Figure 5(a). Under the same dosing, the enrofloxacin-composite nanosystems showed obvious advantages after the second day of administration. Its therapeutic window area was obvious smaller than that of enrofloxacin injection group and enrofloxacin polymeric nanoparticles group. After the end of the experiment, the wound size in the enrofloxacin composite nanosystem group was decreased from the initial 0.50 cm to 0.43 cm, while the wound size in enrofloxacin polymer nanoparticles and enrofloxacin solution groups was almost unchanged and the size in normal saline group was expanded to 0.60 cm (Figure 5b). This may be mainly due to enhanced targeting delivery performance of the composite nanosystems. Besides, the inherent antibacterial activity of CS would enhance the antibacterial effect. Moreover, CS and HA can promote tissue regeneration, allowing cells to grow, proliferate and differentiate (Valachová et al., 2022). At the same time, the colony count of infected sites in mice was carried out. Compared with the untreatment control group, the colony count of enrofloxacin-composite nanosystems, enrofloxacin-polymeric nanoparticles and enrofloxacin injection group after consecutive three day treatment was decreased significantly (Figure 5c). After 3 d post the drug administration, the wound healing and bacterial colony count of in enrofloxacin composite nanosystems group still showed significant advantages compared with enrofloxacin polymeric nanoparticles and enrofloxacin injection group. This may be closely related to its slow-release and target delivery performance. The results showed that the enrofloxacin-composite nanosystems with excellent sustained-release effect and targeting could not only show good antibacterial activity *in vivo*, but also promote wound healing.

## 4. Conclusion

The novel targeted ‘on-demand’ delivery enrofloxacin-composite nanosystem can stay in the body for up to 72 h with no obvious burst release and specifically target bacterial infection sites *in vivo.* Compared with enrofloxacin injection and enrofloxacin polymeric nanoparticles, it significantly inhibited the growth of *S.aureus* and promoted wound healing in mice. Therefore, the enrofloxacin-composite nanosystems may be suitable a candidate for the target treatment of *S. aureus* infections. The composite nanosystem may serve as an effective strategy to overcome the challenges of *S. aureus* and other bacterial infections.
